# Treatment of Iron Deficiency in Heart Failure

**DOI:** 10.1007/s11886-023-01889-4

**Published:** 2023-06-17

**Authors:** Aamir Shamsi, Antonio Cannata, Susan Piper, Daniel I. Bromage, Theresa A. McDonagh

**Affiliations:** 1 Department of Cardiology, King’s College Hospital London, Denmark Hill, Brixton, London, SE5 9RS UK; 2 School of Cardiovascular Medicine and Sciences, King’s College London British Heart Foundation Centre of Excellence, James Black Centre, 125 Coldharbour Lane, London, SE5 9NU UK

**Keywords:** Heart failure, Iron deficiency, Anaemia, ID, Treatment

## Abstract

**Purpose of Review:**

Heart failure (HF) is commonly associated with iron deficiency (ID), defined as insufficient levels of iron to meet physiological demands. ID’s association with anaemia is well understood but it is increasingly recognised as an important comorbidity in HF, even in the absence of anaemia. This review summarises contemporary evidence for the measurement and treatment of ID, in both HFrEF and HFpEF, and specific HF aetiologies, and highlights important gaps in the evidence-base.

**Recent Findings:**

ID is common among patients with HF and associated with increased morbidity and mortality. Correcting ID in patients with HF can impact upon functional status, exercise tolerance, symptoms, and overall quality of life, irrespective of anaemia status.

**Summary:**

ID is a modifiable comorbidity in HF. Therefore, recognising and treating ID has emerging therapeutic potential and is important for all clinicians who care for patients with HF to understand the rationale and approach to treatment.

## Introduction

Iron deficiency (ID) is defined as insufficient levels of iron to meet physiological demands. ID is increasingly recognised as an important phenomenon in heart failure (HF). There is a high prevalence of ID among HF patients, which can impact upon both symptoms and quality of life. Consequently, recognising and treating ID has been shown to be beneficial for patients.

Our understanding of the pathophysiology and treatment of ID in HF is based on the overarching clinical HF phenotype rather than on specific HF aetiologies. This review summarises contemporary evidence for the measurement and treatment of ID in HF and the available evidence for the management of ID in specific HF aetiologies.

## Iron Metabolism

**Fig. 1:**
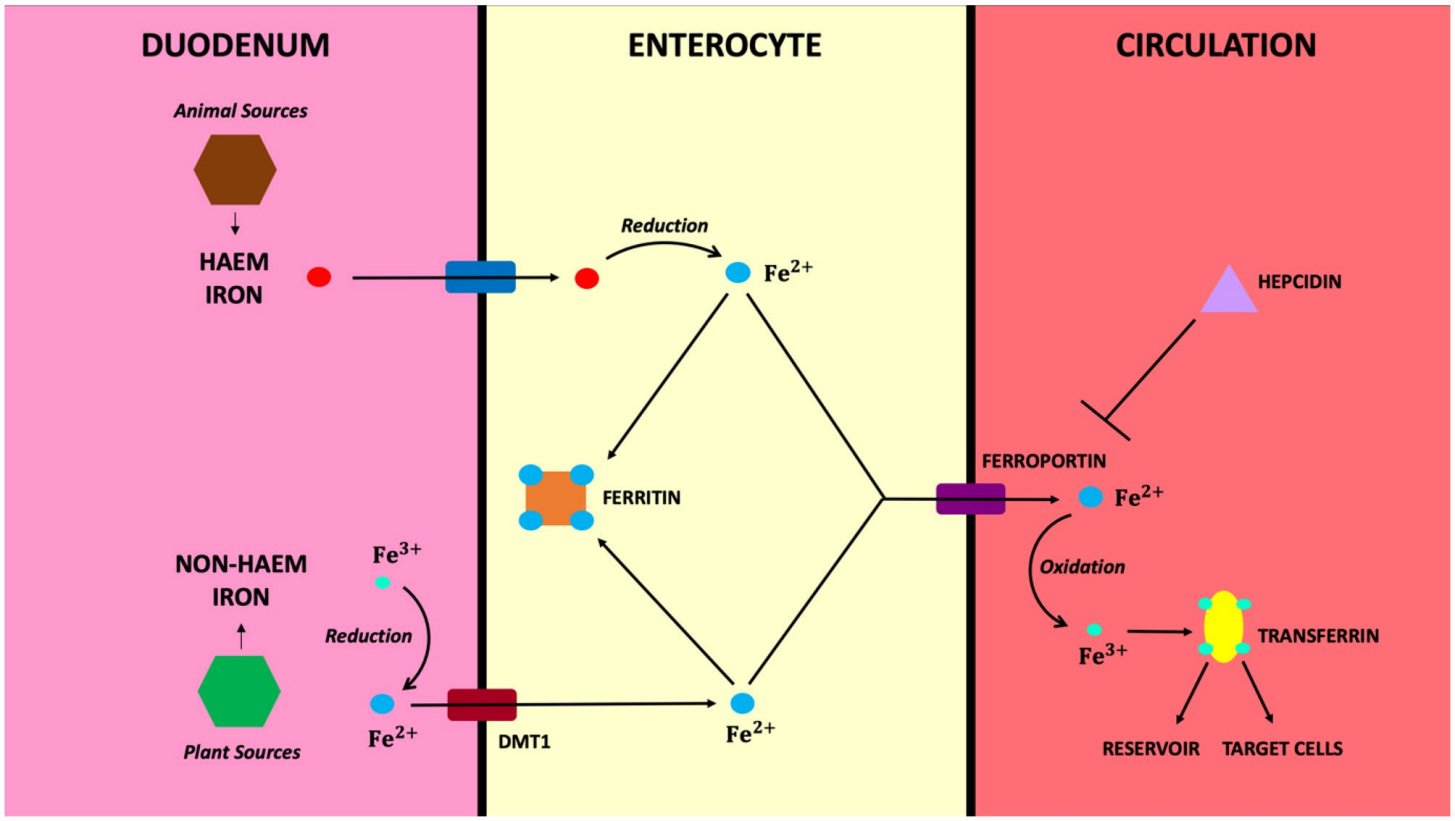
Absorption of iron within the gut and transportation to the circulation

Iron is obtained from our diet and absorbed in the gut. There are two major forms: haem iron, a chelated form of ferrous iron (Fe^2+^) which is derived from haemoglobin and myoglobin and is typically from animal sources, and non-haem iron, or ferric iron (Fe^3+^), that typically originates from plants. Haem iron is absorbed into the enterocyte through several transporters but three in particular have been shown to be particularly important in haem homeostasis: haem carrier protein 1 (HCP1), haem responsive gene 1 (HRG-1), and feline leukaemia virus subgroup C receptor 2 (FLVCR2) [1]. It is then reduced to unchelated ferrous iron [2]. Non-haem iron first undergoes reduction in the gut, as it cannot be directly absorbed, into ferrous iron, before being transported into the enterocyte via divalent metal transporter 1 (DMT1) which is highly expressed in the duodenum in iron deficiency (Fig. 1) [2, 3]. Ferrous iron is toxic within cells and so either needs to be stored in the non-toxic form of ferritin or exported out of the cell via ferroportin. In the circulation, the ferrous iron is oxidised into the ferric form and bound by transferrin as free iron can be harmful outside cells due to its pro-oxidant effect [4]. It then either remains as an iron reservoir or is delivered to target cells [5].

Iron plays a vital role in cellular homeostasis. Some of its pleiotropic roles include being a component of haemoglobin and myoglobin contributing to the transportation and storage of oxygen, acting as an important cofactor for enzymes and proteins involved in oxidative metabolic processes, a role in microRNA biogenesis, contributing to the function of the central nervous and immune systems, and involvement in the synthesis and degradation of proteins, lipids, carbohydrates, DNA, and RNA [6]. As a result, iron is in constant use by high-energy demanding cells in the body.

## Definitions of ID

ID is independent of anaemia and can manifest itself as either low iron store concentrations, called absolute iron deficiency, or insufficient iron supply to meet physiological demands, which is known as functional iron deficiency [7]. Absolute iron deficiency is usually defined as a serum ferritin concentration of < 30 μg/L as ferritin is directly proportional to iron stores [8]. However, given that ferritin is also an acute phase protein, in inflammatory conditions such as HF, it is arbitrarily defined as < 100 μg/L. Transferrin saturation indicates the percentage of transferrin that is iron-bound and acts as a biomarker for available iron. Functional iron deficiency is defined as a ferritin concentration of 100–299 μg/L with a transferrin saturation < 20% [9].

These definitions are based on the FAIR-HF trial that used higher parameters of ID with reference to chronic kidney disease (CKD) patients due to the artificial increases in ferritin seen in chronic inflammatory and oxidative stress, commonly seen in HF as well [7, 10]. As a result, ferritin can become an unreliable marker in advanced disease and acute heart failure (AHF) when stress levels are high. In these states, other markers of ID such as soluble transferrin receptor, which increases in ID and is not affected by inflammation, may be more sensitive, but its use as a tool to guide iron replacement has not yet been proven [11–15]. However, CKD has a different pathophysiology to HF, such as associated uraemia-mediated inflammation and decreased production of erythropoietin, and so, it is still not fully clear whether these definitions, based on serum ferritin and transferrin saturation marker levels, are accurate enough to diagnose ID in HF populations [16].

## Epidemiology of ID in HF

ID is one of the most prevalent comorbidities in chronic heart failure (CHF) [7]. Up to 50% of patients with heart failure and reduced ejection fraction (HFrEF) suffer from lD with the prevalence being higher in patients with NYHA Class III and IV than those with NYHA Class I and II [9, 17]. This figure can be even higher in patients with AHF [18]. Interestingly, in these pooled cohorts, the mean age was relatively low, and elderly patients may also have a higher prevalence [5]. Higher levels of inflammatory markers and NT-proBNP have also been associated with higher prevalence of ID [17, 19]. Other risk factors for ID in HF patients include female sex and advanced HF [20]. ID has also been reported in heart failure with preserved ejection fraction (HFpEF) cohorts with a meta-analysis showing a prevalence of up to 59% [21].

## Mechanism of ID in HF

There are many potential mechanisms for ID in HF. Mucosal oedema from fluid overload and reduced intestinal blood flow can result in impaired absorption of iron from the intestine [22]. The chronic inflammatory process in HF can also impair the metabolism of available iron [18]. It has also been shown that HF patients, particularly those with advanced disease, have a reduced nutritional intake, including iron [23].

There can also be disrupted iron uptake due to increased degradation of luminal iron transporters, ferroportin, by hepcidin, an acute phase protein [7]. Hepcidin can also cause sequestration of iron by inhibiting ferroportin-mediated transport of iron out of enterocytes and macrophages [24]. Hepcidin is upregulated by proinflammatory cytokines, high levels of which are seen in patients with HF. Early stages of systolic HF have been associated with high levels of hepcidin but as the clinical severity developed, hepcidin levels decreased [25]. This paradox remains unexplained but, overall, HF patients typically have elevated levels of hepcidin [25]. As a regulator of iron homeostasis, hepcidin levels might be the most immediate indicator of iron deficiency [26]. However, due to questions over analytical validity as well as cost and confounding factors, it is not currently used as a biomarker for iron status [26]. Iron can also be lost through proteinuria from concomitant chronic kidney disease [27]. In addition, antiplatelet use in underlying ischaemic heart disease can predispose HF patients to gastritis and intestinal ulceration that can lead to iron loss through bleeding [27].

## Importance of ID in HF

Iron has a central role in erythropoiesis, and when iron supplies are low, it can result in anaemia. Anaemia is independently associated with increased mortality and hospitalisation in both HFrEF and HFpEF, with higher risk with more severe anaemia [28]. This is likely to relate to reduced oxygen delivery to the myocardium resulting in increased workload and remodelling [29]. Anaemia is also associated with comorbidities such as kidney disease, which can further worsen outcomes [30].

However, iron also plays an important role in other cellular activities as a co-factor in both haem and non-haem proteins. As a component of myoglobin, iron is involved in oxygen storage, and, via oxidative enzymes and mitochondrial chain proteins, iron is used in the generation of energy for skeletal and cardiac myocytes [7]. It is also used in the synthesis and degradation of carbohydrates, lipids, and nucleic acids [31].

As a result of the wide range of cellular functionals of iron, its deficiency can present clinically in a variety of ways such as fatigue, generalised weakness, or pallor. It can also manifest as dyspnoea, palpitations, or chest pain, especially on exertion, headaches, and lethargy [32]. Hypoxaemia can result in reduced intestinal blood flow causing nausea, abdominal pain, and more critically, malabsorption [32]. Chronic iron deficiency can also result in brittle nails and hair loss.

In HF patients, not only are there low systemic iron levels but also low myocardial concentrations, which likely contribute to the deterioration of cellular function [33]. Multiple observational studies have suggested a strong association of ID with mortality, after adjusting for confounding factors and regardless of anaemia, which further demonstrates the importance of iron on myocardial function and survival [9, 17, 19, 34, 35]. In a prospective observational cohort study of 546 stable systolic HF patients, 3-year survival was 71% in those without ID compared to 59% with ID [17].

Low iron levels have also been associated with exercise intolerance in HF patients due to its impact on cardiac and skeletal muscle. A prospective study of 155 patients with stable HF and an average left ventricular ejection fraction (LVEF) of 26% showed that patients with adequate iron levels had better exercise tolerance than those without, even after the results were adjusted for demographic and clinical variables [36].

Furthermore, low iron levels have also been associated with lower quality of life, irrespective of anaemia status, than those with adequate iron storage supplies in an analysis of > 500 CHF patients [37]. ID is also associated with frailty and is an independent predictor of worse functional capacity [25].

This evidence is predominantly derived from studies of HFrEF. On the contrary, the role of iron and importance of correcting ID in HFpEF is not yet fully understood. An observational study of 751 HF patients found that functional iron deficiency occurred at the same rate in those with and without preserved systolic function [38]. However, less is known about the impact of iron supply on myocardial function, morbidity, and mortality in this group of patients. A multicentre study of 1278 HF patients demonstrated a negative impact on health-related quality of life in those with ID, regardless of whether the LVEF was preserved or reduced [39]. In another small, but well-designed, study of 26 patients with HFpEF, there was no association between cardiac dysfunction and exercise performance with functional iron deficiency [40]. However, a more recent cross-sectional study of 447 HFpEF patients showed that those with ID performed significantly worse in a 6-min walk test than those without and had a worse quality of life [41]. More data is needed in this important group of patients to fully understand the impact of ID.

## Iron Treatment in HFrEF

The association of ID in HF with worse exercise tolerance, hospitalisation, and mortality have resulted in increasing interest in its role in the treatment of HF patients [42], and several therapeutic preparations are available.

### Oral Iron

Oral iron is usually given in the form of ferrous salts such as ferrous fumarate or sulphate. Although it is widely available and inexpensive, absorption of iron through oral administration is low. Furthermore, it can result in common gastrointestinal side effects such as constipation, diarrhoea, and nausea, which can be difficult for patients and result in non-adherence [7, 43]. Animal studies have also shown that ferrous salts can result in adverse outcomes such as intestinal damage through oxidative stress, and long-term cellular iron accumulation can cause necrosis in multiple organs [44, 45]. Therefore, oral iron is not considered a suitable treatment option for ID in those who need a more rapid replacement of iron stores, including those where the rate of chronic loss would surpass the rate of delivery, who are unable to tolerate the side effects, or who have defective intestinal absorption [46].

In HF, the IRONOUT-HF trial randomised 225 HFrEF and iron-deficient patients to either oral iron polysaccharide or placebo [47]. At 16 weeks, there was no difference in peak VO_2_ or 6-min walk distance between the two groups. This may be because luminal absorption in HF patients can be limited due to epithelial dysfunction in the gut as a result of mucosal oedema and reduced intestinal blood flow [48, 49]. In fact, as little as 5% of oral iron is absorbed in HF patients [49, 50]. In addition, because of hepcidin, luminal iron transporters are reduced, and transportation of iron from the gut is inhibited in HF. This is supported by the finding that a subset of patients in IRONOUT-HF who had low hepcidin levels (< 6.6 ng/mL) did increase their iron indices and may benefit from oral iron [47].

There are no other large, randomised trials for oral iron in HF. However, a non-randomised, prospective pilot study showed oral Sucrosomial iron, which has enhanced bioavailability and tolerance compared to conventional oral iron, improved quality of life, and exercise capacity up to 6 months in HFrEF patients with ID [51].

There have not been any large randomised controlled trials comparing oral with intravenous (IV) iron in HFrEF and ID. Only one study, IRON-HF, has attempted this but was terminated due to lack of funding. Twenty-three patients were randomised to either IV iron sucrose, oral ferrous sulphate for 8 weeks or placebo but follow-up data was only available for 18 patients and no significant differences between groups were identified [52]. Preliminary results showed an increase in ferritin and transferrin saturation in both iron groups but superiority of IV iron in iron availability, as measured by TSAT, and improving functional capacity [52]. The IVOFER-HF trial [53], which will compare IV FCM versus oral Sucrosomial iron, in improving exercise tolerance in HFrEF patients with ID will shed more light on this question.

### Intravenous Iron

IV iron administration results in more iron absorption, including in patients with high hepcidin levels [54, 55]. Due to the different preparations of IV iron having different properties, the amount of administered iron can vary, as can the degree of oxidative stress and inflammation from labile plasma iron generation.

First-generation IV iron preparations, such as iron dextran, should be avoided due to a higher risk of anaphylaxis compared to contemporary formulations [56]. Second-generation preparations, such as iron sucrose, are efficient but can only be administered at low doses [56]. Iron sucrose can cause oxidative stress but studies have only been conducted in patients undergoing haemodialysis, which itself causes oxidative stress, inflammation, and endothelial dysfunction [57, 58]. More thermodynamically stable 3rd-generation formulations, such as ferric carboxymaltose (FCM) and ferric derisomaltose (FDI), have shown lower reduction potential and therefore permit higher doses to be administered [59]. In a rat model, FCM was associated with higher oxidative stress levels, a less favourable safety profile and deranged iron deposition when compared to iron sucrose [60]. However, in a randomised single-centre study of non-dialysis-dependent CKD patients, 3rd-generation IV iron showed good safety profiles and no oxidative stress, with FDI giving more efficient iron repletion [61].

IV iron introduces large amounts of non-transferrin-bound iron, which bypasses regulatory mechanisms, including hepcidin, and can cause iron overload which can be cardiotoxic [16]. Infusion of ferric saccharate can lead to acute endothelial dysfunction and increased oxygen radical stress in healthy individuals [62]. Therefore, it is important to carefully regulate the intake and required amount of therapy. Finally, caution should be exercised in acute infection. Data from observational studies in haemodialysis patients have been conflicting but the consensus is to avoid IV iron in active systemic infections due to the risk of impairing neutrophil and T-cell function [63, 64].

In HF patients, single or periodic boluses of IV iron may be preferable to regular oral iron, and several studies have investigated its utility in this setting (Table 1). In a small study of 35 CHF patients with anaemia, there was an increase in exercise capacity and improvement of symptoms in patients randomised to iron treatment, compared to no treatment, with NYHA function class also improving in non-anaemic patients receiving iron [65]. In another small, double-blind study of 40 CHF patients with ID that were randomised to either IV iron sucrose therapy or placebo for 5 weeks showed that at 6 months, 1 year, and 5 years, the number of hospitalisations was significantly less frequent in the IV iron group compared to placebo, and at 5 years, mortality was also reduced [66, 67]. The Myocardial-IRON trial was a multicentre double-blinded study looking at 53 HF, and iron-deficient patients who received IV FCM showed that they had associated T2 and T1 mapping changes on cardiac MRI in keeping with myocardial iron repletion [68].

**Table 1:** Summary of the major current and planned randomised trials looking at IV and oral iron in HFrEF and HFpEF

Trial	Patient group	Design and iron preparation	Outcome
HFrEF trials			
Tobli et al. [66, 67]	ID patients with Hb < 12.5 g/dL, CrCl < 90 mL/min, and LVEF ≤ 35%	Double-blinded study with 40 patients randomised to placebo or IV iron sucrose complex for 5 weeks. Primary endpoints were changes in renal and haematological parameters, NT-proBNP and CRP levels at 6 months	IV iron showed better haematological values, renal function, ↓NT-proBNP, and ↓CRP. LVEF also improved as did symptoms, measured by 6MWT, and ↓hospitalisations at 6-month, 1-year, and 5-year follow-up. Mortality at 5 years was also significantly lower with IV iron
FERRIC-HF [65]	ID and CHF patients with LVEF ≤ 45%, NYHA Class II-III, Hb ≤ 14.5 g/dL, and pVO2 14.0 ± 2.7 mL/kg/min	Randomised 35 patients to receive 16 weeks of either IV iron sucrose or no treatment. The observer-blinded primary end point was the change in absolute pVO2	IV iron improved NYHA class (*p* = 0.007) and pVO2/kg (*p* = 0.01). In anaemic patients, there was an improvement in absolute pVO2 (*p* = 0.02) and pVO2/kg (*p* = 0.01) with IV iron. Adverse events were similar between two groups
FAIR-HF [10]	CHF patients with ID, LVEF ≤ 40% for NYHA II patients, and LVEF ≤ 45% for NYHA III patients, Hb 9.5–13.5 g/dL	459 patients were randomly assigned, in a 2:1 ratio, to IV FCM or placebo. The primary end points were the self-reported PGA and NYHA functional class at week 24	47% of IV FCM vs. 30% placebo patients had NYHA functional class I or II at week 24 (OR 2.40; 95% CI, 1.55 to 3.71). Results similar with and without anaemia. IV FCM ↑distance on 6MWT and QoL assessments. The rates of death, adverse events, and serious adverse events were similar in the two groups
IRON-HF [52]	Patients with LVEF < 40%, NYHA class II-IV, Hb 9–12 g/dL, Tsat < 20%, and ferritin < 500 μg/L	Double-blinded multicentre trial of 18 patients randomised to IV iron sucrose for 5 weeks, oral ferrous sulphate for 8 weeks or placebo. Primary endpoint was variation of pVO2 over 3-month follow-up	Those treated with IV iron had ↑ functional capacity (increase of 3.5 mL/kg/min in pVO2). ↑Ferritin, ↑TSat, and ↑Hb in both treated groups
CONFIRM-HF [69]	Iron-deficient patients with stable CHF, LVEF ≤ 45% M NYHA II-III on optimal background therapy for CHF	304 patients were randomised 1:1 to IV FCM or placebo for 52 weeks. The primary end-point was the change in 6MWT distance from baseline to week 24	With IV FCM ↑6MWT distance at week 24 (*p* = 0.002), this was consistent among all subgroups and sustained to week 52. Also, ↑NYHA class, ↑PGA, and ↑QoL in patients treated with IV FCM from week 24 onwards. Treatment with IV FCM ↓hospitalizations for worsening HF (HR 0.39 (0.19–0.82), *p* = 0.009). Death rate and the incidence of adverse events comparable between both groups
IRONOUT-HF [47]	Patients with HFrEF (< 40%) and ID	Double-blind trial of 225 patients randomised to receive either oral iron polysaccharide or placebo for 16 weeks. The primary end point was a change in pV̇O2 from baseline	The change in pV̇O2 and 6MWT at 16 weeks did not significantly differ between the oral iron and placebo groups
EFFECT-HF [70]	ID subjects with stable CHF, LVEF ≤ 45%, NYHA II–III, and on optimal background therapy	172 patients randomised to treatment with IV FCM for 24 weeks or standard of care. The primary end point was the change in pVO_2_ from baseline to 24 weeks	IV FCM significantly ↑ serum ferritin and transferrin saturation. At 24 weeks, ↓pVO_2_ in control group but maintained on IV FCM. ↑PGA and ↑NYHA improved on IV FCM versus standard of care
Myocardial-IRON [68]	Patients with ID, LVEF < 50%, NYHA II–III symptoms and Hb < 15 g/dL	Multicentre double-blinded study. 53 patients randomised to IV FCM or placebo. Primary objective was to assess myocardial iron repletion estimated by T2* and T1 mapping CMR sequences 7 and 30 days after treatment	Baseline T2* and T1 mapping values did not significantly differ across treatment arms. On day 7, both T2* and T1 mappings were significantly lower in the FCM arm (*p* = 0.025) indicating repletion. At 30 days, there was a similar reduction on T2* mapping (*p* = 0.003) but not T1
AFFIRM-AHF [71••]	Patients with ID and admitted with acute HF with LVEF < 50%	1108 participants randomised to IV FCM or placebo for up to 24 weeks, dosed according to the extent of iron deficiency. The primary outcome was a composite of total hospitalisations for heart failure and CV death up to 52 weeks	370 total CV hospitalisations and deaths occurred in IV FCM group and 451 in placebo group (RR 0.80, 95% CI 0.64–1.00, *p* = 0.050). No difference in CV death between groups. 217 HF hospitalisations occurred in FCM group and 294 in placebo group (RR 0.74; 95% CI 0.58–0.94, *p* = 0.013)
IRONMAN [72••]	ID patients with new or established HFrEF (LVEF ≤ 45%) and current or recent (within 6 months) hospitalisation for HF	Prospective, randomised open-label, blinded endpoint (PROBE) event-driven. Iron preparation is IV ferric derisomaltose. Primary endpoint was combined rate of recurrent hospitalisations for HF and CV death	336 primary endpoints in the IV ferric derisomaltose group and 441 in the usual care group (RR 0.82, 95% CI 0.66–1.02, *p* = 0.070). In the prespecified COVID-19 sensitivity analysis, 210 primary endpoints in the IV ferric derisomaltose group and 280 in the usual care group (RR 0.76, 95% CI 0.58–1.00, *p* = 0.047), mainly driven by a reduction in HF admissions
HEART-FID [73] NCT03037931	Stable CHF patients with ID, Hb 9–13 g/dL (females) or < 15 g/dL (males) and LVEF ≤ 40% on optimal therapy	Multi-centre and double-blinded study with patients randomised to IV FCM or placebo. Assessing 12-month rate of death, hospitalisation for worsening HF, and the 6-month change in 6MWT distance	Estimated to end June 2023
FAIR-HF2 [74] NCT03036462	HFrEF patients for at least 12 months, iron deficiency, Hb 9.5–14 g/dL. Estimated enrolment of 1200 patients	Multi-centre and double blinded study with patients randomised to IV FCM or placebo. Primary endpoint of combined rate of recurrent HF hospitalisations and CV death in 12 months	Estimated to end May 2024
IVOFER-HF [53] 2017–005,053-37	HFrEF (LVEF ≤ 45%) patients on optimal medical therapy with NYHA II-IV class symptoms and ID	Patients randomised to IV FCM, oral iron (ferrous sulphate or liposomal iron) or placebo. Primary endpoint is a change in 6MWT distance from baseline to week 24	Unknown end date
HFpEF trials			
FAIR-HFpEF [75] NCT03074591	Patients with HFpEF (LVEF ≥ 45%) and ID, with or without anaemia, Hb 9.0–14.0 g/dL and recent HF hospitalisation	Patients will be randomised to IV FCM or placebo. Primary outcome is difference in 6MWT from baseline to end of study between two groups	Unknown end date
PREFER-HF [76] NCT03833336	Stable CHF patients on optimal medical therapy with ID and LVEF > 45%	Randomised trial with quadruple masking. Patients randomised to placebo, IV FCM, oral ferroglycine sulphate, or oral Sucrosomial iron. Primary outcome is change in 6MWT from baseline to week 24	Unknown end date

*ID* iron deficiency, *Hb* haemoglobin, *CrCl* creatinine clearance, *LVEF* left ventricular ejection fraction, *IV* intravenous, *NT-proBNP* N-terminal pro B-type natriuretic peptide, *CRP* C-reactive protein, 6MWT 6-min walk test, *CHF* chronic heart failure, *NYHA* New York Heart Association functional classification, *pVO2* peak oxygen consumption, *FCM* ferric carboxymaltose, *PGA* patient global assessment, *QoL* quality of life, *TSat *transferrin saturation, *HF* heart failure, *HFrEF* heart failure with reduced ejection fraction, *CMR* cardiac magnetic resonance imaging, *CV* cardiovascular, *HFpEF* heart failure with preserved ejection fraction

The use of IV FCM has been shown to be both safe and to improve symptoms, exercise capacity, and quality of life independent of anaemia [18]. In the FAIR-HF trial, a randomised and double-blind study of 459 patients with CHF, NYHA class II and LVEF < 40% or class III and LVEF < 45%, ID and a haemoglobin (Hb) of 95–135 g/L, and treatment with IV FCM at a dose of 200 mg of iron improved symptoms and quality of life from weeks 4 to 24 with no difference in mortality or adverse events [10]. NYHA class improved in 47% of patients receiving IV iron compared to 30% receiving placebo, and there was also a greater improvement in the 6-min walk test [10]. The median ferritin level was 39 μg/L in this population. These beneficial effects of IV iron were observed in patients despite baseline anaemia status, LVEF, Hb level, or NYHA class [10].

The multi-centre and double-blinded CONFIRM-HF trial of 304 patients showed that in patients with LVEF < 45% in NYHA Class II-III and ID with Hb < 15 g/dL, there was a significant improvement in quality of life, functional capacity, and symptoms 24 weeks after treatment with IV FCM, at a total dose of 500–2000 mg [69]. This effect lasted up to a year as well as finding a reduced risk of HF hospitalisation. The median ferritin level was 46 μg/L in this population group. The benefits of IV iron were seen in all subpopulations at week 24 irrespective of baseline NYHA class, Hb, LVEF, and ferritin levels [69]. Similar to FAIR-HF, the rate of death and adverse events in both arms were equivalent [69]. In CONFIRM-HF, patients were followed up for a year as opposed to 6 months in FAIR-HF and were given higher doses of IV iron. This finding was supported by the subsequent EFFECT-HF study of 172 patients, which showed that IV FCM in symptomatic HF patients increased iron store levels and potentially exercise capacity after adjustment with imputation strategy for patients that died [70].

AFFIRM-AHF was a multicentre double blinded trial looking at 1108 patients hospitalised with AHF who had ID and LVEF < 50%. Patients were randomised to receive either placebo or IV FCM, repeated at 6- and 12-week intervals. IV FCM reduced the composite primary endpoint of first HF hospitalisation or cardiovascular death, with a *p* value just short of significance (0.059), but did improve total HF hospitalisations up to 52 weeks post treatment. There was no effect on the risk of cardiovascular death [71••]. It did, however, improve health-related quality of life as early as 4 weeks after initiation [77].

The recently reported IRONMAN study was a large, randomised outcome trial of ferric derisomaltose compared to usual care in 1137 UK patients with LVEF ≤ 45%, transferrin saturation < 20%, or serum ferritin < 100 μg/L [72••]. At a median follow-up of 2.7 years, the primary outcome of recurrent hospital admissions for HF and cardiovascular death was reduced by 18% (*p* = 0.070). In the prespecified COVID-19 sensitivity analysis, the reduction in the primary endpoint was statistically significant (*p* = 0.047), which was driven by a reduction in HF admissions. It was therefore consistent with AFFIRM-AHF, finding no effect on cardiovascular mortality [78].

A recently published meta-analysis of randomised controlled trials, not including IRONMAN, evaluating the effect of IV iron in 851 patients with systolic HF and ID, showed a reduction in the combined end-point of all-cause mortality or cardiovascular hospitalisation, cardiovascular death, or hospitalisation due to worsening HF as well as an increase in exercise capacity and quality of life [79].

Ongoing trials include FAIR-HF2 (NCT03036462), which is investigating hospitalisation and cardiovascular death in iron deficient HF patients receiving a maximum of 2000 mg FCM within 4 weeks followed by 500 mg every 4 months [74] and HEART-FID (NCT03037931) [73], investigating IV FCM on incidence of death and hospitalisations.

## Iron Treatment and Cardiac Resynchronization Therapy

As well as improving cardiac myocyte health, one of the proposed methods by which iron repletion improves functional status and exercise tolerance could be its beneficial impact on ventricular remodelling and improving systolic function [80]. The RIDE-CRT study showed that ID was a negative predictor of effective cardiac resynchronization therapy (CRT) due to reverse cardiac remodelling [81]. The recent randomised, double-blind IRON-CRT trial showed that patients with ID that had persistently reduced LVEF < 45% 6 months after CRT implant benefitted from IV FCM as measured by increased LVEF, functional capacity and cardiac force-frequency relationship, and a decrease in LVESV at 3 months [82]. Interestingly, treatment with FCM also improved right ventricular function and contractile reserve [83].

## Iron Therapy and HFpEF

The focus of IV iron therapy has been on HFrEF patients as no formal randomised controlled trials have yet evaluated IV iron in patients with HFpEF and ID. However, a retrospective single-centre study showed there was an increase in ferritin, TSAT, and haemoglobin in stable HFrEF and HFpEF patients who were given IV FCM as well as an improvement of functional status between the two groups [84]. Large, randomised trials in this patient population are required to ascertain the benefit of IV iron as a treatment for ID. The FAIR-HF-HFpEF study [75] is looking at the impact on exercise tolerance of FCM vs. placebo in ID patients with LVEF > 45%, and PREFER-HF [76] is looking at the effect of both oral and intravenous iron on symptoms and functional class.

## Guideline-Directed Management of ID in HF

**Fig. 2:**
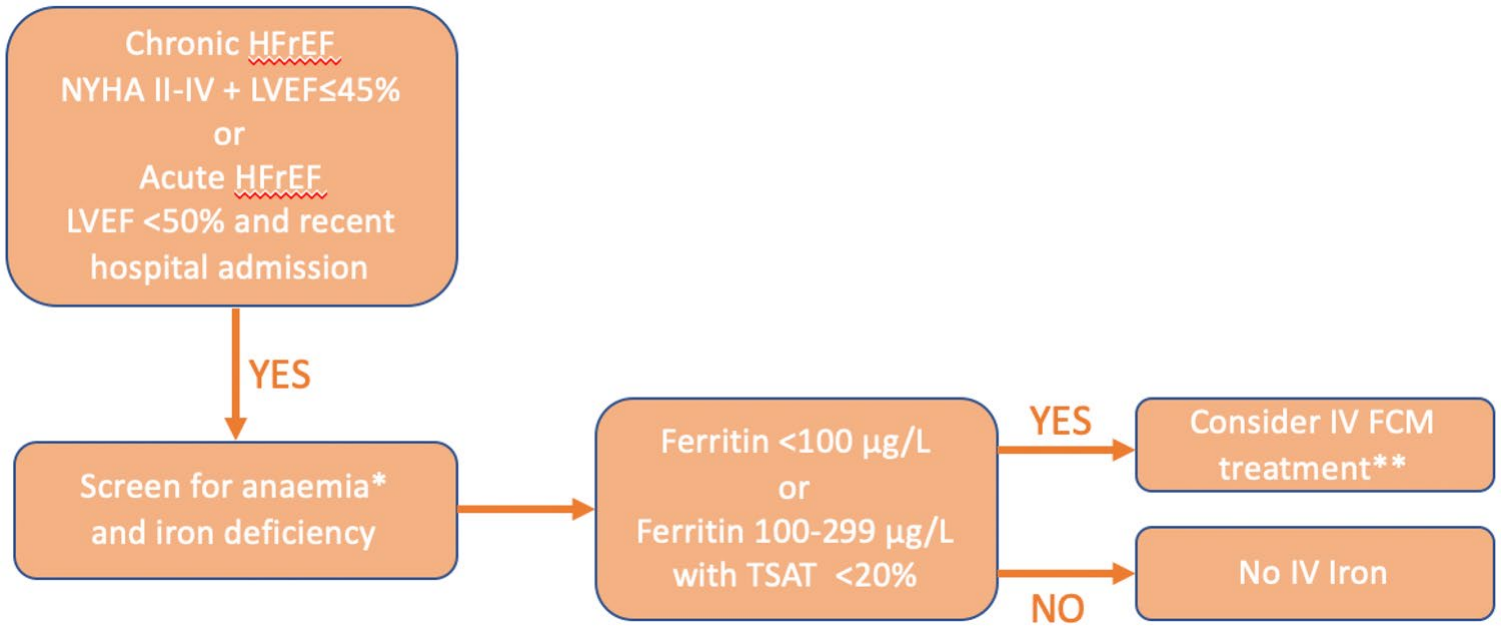
A summary of the diagnosis and treatment of iron deficiency in heart failure. *If significant anaemia < 9 g/dL, then consider, investigate, and treat alternative causes for anaemia other than iron deficiency. **Check contraindications and cautions before treatment. Abbreviations: HFrEF, heart failure with reduced ejection fraction; NYHA, New York Heart Association; LVEF, left ventricle ejection fraction; TSAT, transferrin saturation; IV, intravenous; FCM, ferric carboxymaltose

ID is a common comorbidity in HF patients, irrespective of anaemia status, and can result in reduced exercise tolerance, recurrent decompensation of HF, and increased cardiovascular and all-cause mortality [85, 86]. The 2021 European Society of Cardiology (ESC) Guidelines for the Diagnosis and Treatment of Acute and Chronic Heart Failure states that all HF patients should be regularly screened for anaemia and ID, and detection of either should prompt investigation and treatment [18]. Based on the available data, it also recommends that IV iron supplementation with FCM should be considered to alleviate HF symptoms and improve exercise tolerance or quality of life in patients with ID, defined as above, and symptomatic HF with LVEF < 45% or LVEF < 50% with a recent HF hospitalisation [18]. The American guidelines, published in 2022, recommended that all HF patients should have iron studies as part of their initial assessment and suggested that IV iron replacement in patients with HFrEF and ID is reasonable to improve functional status and quality of life as a class IIa recommendation, but gave no recommendation on a specific formulation [87]. A summary of the diagnosis and treatment of ID in HFrEF based on guidelines and evidence presented can be found in Fig. 2.

Patients should be made aware of the common side effects that include headache, dizziness, and injection site reactions [5]. Anaphylaxis is rare with 3rd-generation IV iron preparations, but it should always be administered in a clinical setting with access to a resuscitation team. It should also be avoided in patients with active bacteraemia [5]. Oral iron is not recommended in the treatment of ID in systolic HF patients as it has neither been shown to be affective in iron repletion nor to improve exercise tolerance or health status [47]. However, oral Sucrosomial iron, which has shown similar efficacy to IV iron in some conditions associated with ID, could be a promising area in the future [51].

McDonagh et al. suggested an algorithm for the treatment of ID in HF patients that advises either weekly 200 mg FCM to correct ID followed by 4-weekly maintenance 200 mg doses based on FAIR-HF trial or a single 500–1000 mg dose of FCM and 500 mg maintenance dose thereafter to maintain iron level targets based on CONFIRM-HF [7]. Large trials using other formulations of IV iron are ongoing in HFrEF and AHF populations, which will help guide us on the best management options available.

## Role of ID in Specific HF Aetiologies

It is not known if there are important differences in the pathophysiology, treatment, and outcomes of ID in different aetiologies of HF as the available studies, described in this review, are not adequately powered to answer this question. It has been shown that ID in both non-ischaemic dilated cardiomyopathy (DCM) and ischaemic cardiomyopathy (ICM) end-stage HF patients resulted in dysfunctional mitochondria and increased oxidative stress in the left ventricle which then drove pathological remodelling of the heart and worsening of LVEF and NYHA classification [88], but no comparison to other aetiologies was performed. However, in AFFIRM-HF, patients with non-ischaemic HF aetiologies did not benefit from IV iron in relation to the primary endpoint of recurrent HF hospitalisations and cardiovascular death compared to ischaemic aetiologies (RR 1.11 vs. 0.6) [71••], which is a hypothesis-generating finding. No other trials have distinguished between aetiologies. Furthermore, most trials of IV iron excluded patients with recent acute coronary syndrome or cardiac surgery and those with uncorrected significant valvular heart disease, which means the impact of IV iron in these HF subgroups remains unclear. Possible differential effects of iron therapy in different HF aetiologies remains unanswered and is an interesting area for further research.

## Conclusions

ID is a common comorbidity in HF patients and has an increasingly understood role in its morbidity and mortality. The mechanism of ID in HF patients varies from reduced absorption, impaired uptake, metabolism, and transportation of available iron as well as loss. Multiple studies have investigated IV iron in HF patients, mainly focusing on HFrEF. To date, large randomised controlled trials have shown a clear benefit in correcting ID, irrespective of anaemia status, in chronic HF patients with IV iron, with 3rd-generation preparations such as FCM, with respect to improving symptoms, functional status, exercise tolerance, reducing hospitalisations, and quality of life. However, discernible impact on mortality has not yet been demonstrated, and long-term data on safety is required. More trials using different iron preparations, including newer oral formulations, in varying clinical settings such as HFpEF and AHF are awaited. More research is required in other key areas. We need more data on whether the beneficial impact of IV iron is linked to certain aetiologies of HF. Furthermore, the definition of ID in HF is still debatable and newer biomarkers such as hepcidin, and soluble transferring receptor may hold the key for more accurate diagnosis of ID. We also need a better understanding of the long-term potential damaging consequences of IV iron, particularly relating to oxidative stress and endothelial function. With ID becoming much more topical, we will gain a more robust appreciation of its impact and how we can best treat it in HF.

## Data Availability

This paper does not include any original data. All data referred to in the text has been referenced.
